# Delayed, Progressive Multivessel Occlusion After Resection of a Recurrent Glioma

**DOI:** 10.7759/cureus.33019

**Published:** 2022-12-27

**Authors:** Onur Tanglay, Nicholas B Dadario, Isabella M Young, Jacky T Yeung, Charles Teo, Michael E Sughrue

**Affiliations:** 1 Department of Neurosurgery, Prince of Wales Private Hospital, Sydney, AUS; 2 Department of Neurosurgery, Robert Wood Johnson Medical School, Rutgers University, New Brunswick, USA; 3 Research, Omniscient Neurotechnology, Sydney, AUS; 4 Department of Neurosurgery, Yale University, New Haven, USA

**Keywords:** glioblastoma, resection, glioma, ischemia, complication

## Abstract

Glioblastoma multiforme (GBM) is one of the most common primary brain tumors with an aggressive natural history consistent with a median survival of less than two years. Most clinical research has primarily focused on improving overall survival through aggressive cytoreductive surgery and adjuvant radiochemotherapy. However, far less clinical guidance has been given for unexpected instances of neurologic decline following safe glioma resection in the setting of vascular etiology. Here, we report a 50-year-old man who presented to our clinic with a seizure. His preoperative magnetic resonance imaging (MRI) demonstrated a left hippocampal glioblastoma. Ten months following total resection, the patient presented again with rapid loss of vision and hemorrhagic papilledema. An MRI demonstrated a recurrence of his glioma, which was partially resected with no complications. Eight days after surgery, the patient suddenly became unresponsive and imaging revealed moderate blood in the resection cavity, which was evacuated in the operating room. Follow-up scans showed a posterior cerebral artery infarction, and two days later, a middle cerebral artery infarction, upon which care was withdrawn. We do not propose a mechanism by which this delayed ischemia occurred, especially as the middle cerebral artery was not damaged during surgery, however, we note that delayed ischemia may be one mechanism of damage following glioma resection, which should be studied further to improve patient outcomes.

## Introduction

It is well-established that glioblastoma confers a hypercoagulable state in patients [1-4]. The high rate of deep venous thrombosis is one manifestation of this phenomenon [5]. It is also well-known that ischemic injury can occur following the resection of gliomas [6-8]. While there is nearly always diffusion restriction around the edge of a resection cavity [9], it is far more substantial when, in some cases, the diffusion restriction extends along with vascular territories well outside the field of resection, suggesting loss of arterial vessel patency [10,11]. Most commonly, this is the result of injury to a blood vessel during surgery [10].

While it is known that the events following brain trauma involve a complex set of compensatory, and in some cases, detrimental molecular and cellular cascades in response to the injury [12], far fewer studies have been directed toward the events following resection of gliomas, which differ in the focality of the injury and the presence of tumor tissue in the neighboring brain. The case presented here provides one example of a potentially unique mechanism, namely, the role of delayed vascular ischemia, which may occur in some patients and therefore represents an important area of future study to optimize postoperative outcomes.

## Case presentation

**Figure 1:**
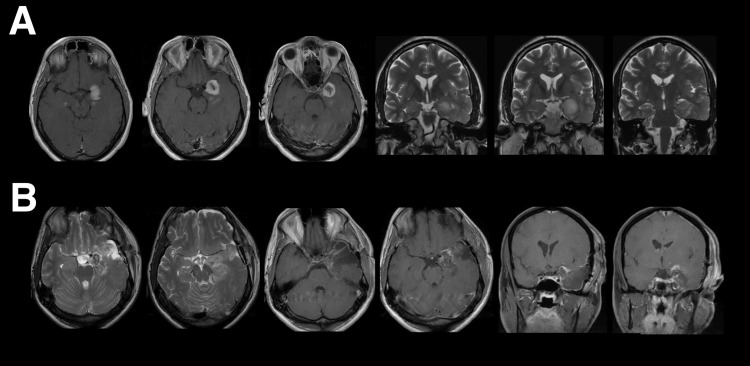
Preoperative and postoperative neuroimaging Preoperative (A) and postoperative (B) axial and coronal gadolinium-enhanced T1 and T2-weighted MR images demonstrating a left hippocampal glioma and the effects of a subsequent total resection.

The patient was a 50-year-old man who presented with a seizure and was subsequently found to have a left hippocampal glioblastoma. The mass was resected in total, and the patient was clinically stable following surgery. Pre- and postoperative imaging can be seen in Figure 1. His follow-up treatment plan included standard adjuvant fractionated radiotherapy and temozolomide per the Stupp protocol [13].

**Figure 2:**
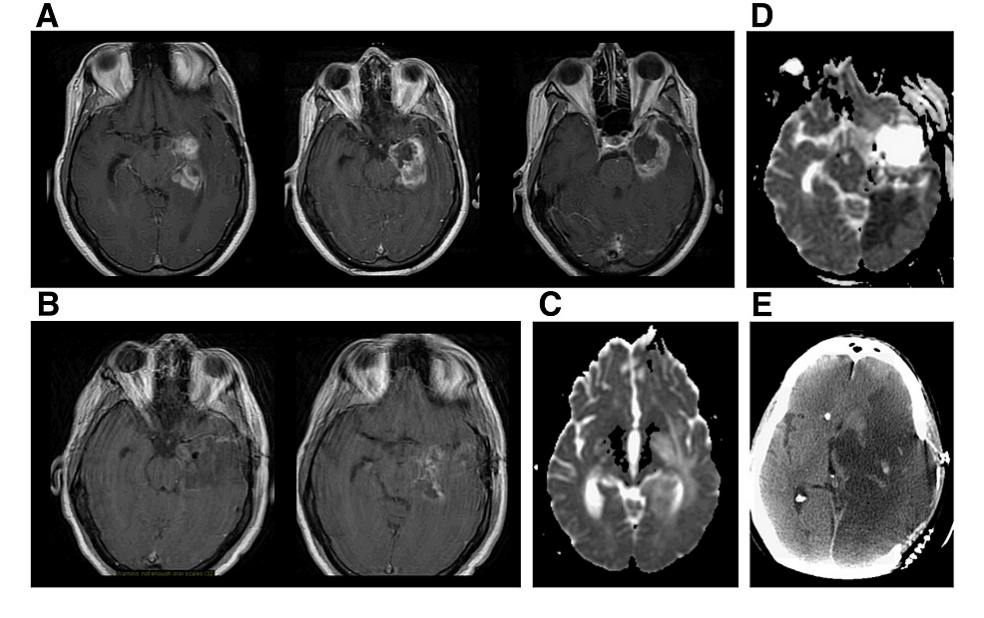
Neuroimaging of delayed ischemia Preoperative (A) and postoperative (B) axial gadolinium-enhanced T1-weighted MR images showing a recurrence of the temporal glioma around the edges of the previous resection cavity and partial resection with a thin layer of tumor remaining in the medial temporal lobe. Postoperative DWI (C) shows no evidence of ischemia. Eight days following surgery, DWI shows acute blood in the resection cavity while CT imaging shows a dense PCA (D). Two days following this scan, imaging revealed a large MCA infarction (E). DWI: diffusion-weighted imaging; PCA: posterior cerebral artery; MCA: middle cerebral artery

He remained well for approximately 10 months until he experienced a rapid loss of his vision over two days without prior warning or previous visual complaints. The patient was examined and found to have hemorrhagic papilledema. His MRI also demonstrated a recurrent tumor around the edges of the previous resection cavity (Figure 2A). The best hypothesis for the rapid decline in his neurologic status was that the tumor was causing increased intracranial pressure. The decision was made to take the patient to the operating room and remove his recurrent tumor. During the procedure, the patient’s brain was not markedly swollen, and we were able to remove all but a thin layer of tumor in the medial temporal lobe (Figure 2B). We did not see or cauterize any vessels medial to the tentorial incisura. The postoperative diffusion-weighted MRI scan did not demonstrate any evidence of ischemic stroke (Figure 2C).

The patient did well initially for eight days after the surgery. He was walking in the hallways and had a normal neurologic exam save for his loss of vision, which was unchanged following surgery. The patient then abruptly became unresponsive while recovering in a rehabilitation facility and was found to have a moderate amount of blood in the resection cavity. Even though it was unclear how a relatively small amount of blood could cause his significant decline, it was decided to take him to the operating room, and we performed an emergency clot evacuation. During that surgery, no active site of bleeding was noted, and no vessels were cauterized. The patient’s follow-up scans showed a left posterior cerebral artery (PCA) infarction, which had developed in the intervening period from the initial postoperative scan (Figure 2D). A monitor placed during the clot evacuation surgery demonstrated no increased intracranial pressure in the immediate postoperative period. Two days later, repeat imaging demonstrated interval development of a dense middle cerebral artery (MCA) stroke (Figure 2E). Subsequently, the patient’s intracranial pressure could not be controlled, and following discussions with the family, care was withdrawn.

Postmortem examination demonstrated only the post-surgical changes and ischemic stroke described. There were no signs of infection during the postoperative period. Cerebrospinal fluid sent during the perioperative period showed no signs of infection following the intracranial hemorrhage, PCA stroke, or MCA stroke.

## Discussion

In this case, we report an example of a patient who underwent an uncomplicated resection of a recurrent temporal glioma in the setting of hemorrhagic papilledema. The patient was initially stable following surgery but proceeded to develop an intracranial hemorrhage into his resection cavity as well as a PCA stroke approximately one week following surgery, which were not present on immediate postoperative scans. The patient went on to expire from an interval MCA stroke.

In cases such as this, it is often difficult to find a satisfactory explanation, yet we may speculate on the underlying cause. One possibility is that the patient developed fulminant, progressive deterioration, with vasculopathy playing a central role. While the hemorrhagic papilledema suggested that the patient had increased intracranial pressure, we have no evidence that his intracranial pressure was ever elevated at any point in his care. Therefore, vascular etiology may be more likely. Indeed, hemorrhagic papilledema has been reported in several cases, including chronic myeloid leukemia, paroxysmal nocturnal hemoglobinuria, iron deficiency anemia, and subarachnoid hemorrhage [14-22]. Given the sequential occlusion of two vessels that were not manipulated during the patient’s second tumor surgery, this raises the possibility of hypercoagulable state-related vessel occlusion or other vasculopathies [23-27].

Most neurologic decline following glioma surgery is attributed to brain swelling [28]. However, this case provides a dramatic example of the possible role of other mechanisms in neurologic decline following glioma resection. The idea that hypercoagulability [29], vasospasm [30], or venous congestion [31] could be exacerbating postoperative ischemia raises important questions about where we might intervene to improve patient outcomes, which require further study to answer.

## Conclusions

This report presents a rare case of an uncomplicated recurrent GBM resection, which resulted in a delayed intracranial hemorrhage into the resection cavity and then a subsequent PCA and MCA stroke one week postoperatively. Further study is necessary to better understand the mechanisms of brain injury following uncomplicated glioma resection, which may result in immediate or delayed postoperative ischemia in order to optimize opportunities for vascular intervention in the perioperative period. In particular, delayed ischemia may occur after glioma resection without obvious iatrogenic injury and therefore demonstrates an unclear mechanism and an important possibility that neurosurgeons should consider in glioma surgery moving forward.
